# Sea Voyages

**Published:** 1858-03

**Authors:** 


### SEA VOYAGES

AND the South, are the universal recommendations for consumptive persons. For many years we have opposed this view, because we had seen the things we spoke of. M. ROCHERA, a surgeon in the French navy, has lately written a prize essay on the subject, and sums up the whole matter thus:

1. Sea voyages much oftener accelerate than retard the progress of consumption.
2. Consumption is more frequent among sailors than among land forces.
3. The progress of consumption is more rapid on board ship than on shore.
4. The naval profession should be strictly interdicted to those who seem to be threatened with consumption.
5. Land journies afford greater facilities for carrying out what is necessary, in any given case, than a sea-voyage, with less danger and less cost.
6. Hot countries accelerate the progress of consumption; and this is the unanimous opinion of the chief surgeons in both French and British colonies.
7. It is only in the very first stages of consumption that we are authorized in expecting good results from a change of climate.

We may well inquire, in this last case, is not that advantage the result of the patient being well enough to be out of doors a great deal, and might not still higher benefits result, if the same amount of out-door activity were practiced at home? See this fully discussed in our Book on "Consumption."

